# High-Concentration Gold Nanoparticle Pastes for Advanced Deposition-Based Sensor Manufacturing

**DOI:** 10.3390/s26113507

**Published:** 2026-06-02

**Authors:** Aleksandra Motyka, Sławomir Drozdek, Nina Szczotka, Iwona Grądzka-Kurzaj, Krzysztof Kubica, Aneta Wiatrowska, Karol Malecha

**Affiliations:** 1 XTPL SA, Legnicka 48E, 54-202 Wrocław, Poland; slawomir.drozdek@xtpl.com (S.D.); nina.szczotka@xtpl.com (N.S.); iwona.gradzka-kurzaj@xtpl.com (I.G.-K.); krzysztof.kubica@xtpl.com (K.K.); aneta.wiatrowska@xtpl.com (A.W.); 2 Institute of Low Temperature and Structure Research, Polish Academy of Sciences, Okólna 2, 50-422 Wrocław, Poland; 3 Department of Physical and Quantum Chemistry, Faculty of Chemistry, Wrocław University of Science and Technology, Wybrzeże Wyspiańskiego 27, 50-370 Wroclaw, Poland; 4 Department of Microsystems, Faculty of Electronics, Photonics and Microsystems, Wrocław University of Science and Technology, Wybrzeże Wyspiańskiego 27, 50-370 Wrocław, Poland

**Keywords:** gold nanoparticles, ultra-precise dispensing technology, gold microsensors and fractals

## Abstract

There is a growing demand for extreme miniaturization and enhanced sensitivity in next-generation sensing systems, including wearable devices and bioelectronics. Such advanced platforms require highly conductive, biocompatible, and mechanically robust architectures capable of conforming to dynamic surfaces. Conventional metallic thin-film fabrication techniques have reached their fundamental physicochemical limits, often suffering from suboptimal mechanical strength, complex multi-step processing, and high costs. In contrast, additive manufacturing methodologies offer streamlined microfabrication, yet traditional printing methods frequently struggle with low-viscosity constraints, insufficient metal loading, and significant material losses. This paper covers the morphological fidelity, mechanical resilience, and electrical performance of rheologically tailored, high-concentration (above 90%) gold nanoparticle paste deposited via Ultra-Precise Dispensing (UPD) technology. The capability of the UPD system to print complex, high-density fractal geometries with linewidths down to 5 μm is evaluated on both rigid and flexible substrates, glass and polyimide, respectively. The mechanical structural integrity of these conductive traces is characterized under initial 360-degree bending tests. Finally, the electrical stability and thermal response of a printed proof-of-concept temperature sensor are evaluated. The printed fractal microstructures exhibit good resolution and the fabricated sensor demonstrates good stability, displaying a linear thermal response with a temperature coefficient of resistance of 1.98·10^−3^ °C^−1^, validating this combined material-deposition approach for microelectronics.

## 1. Introduction

The contemporary digital economy paradigm is intrinsically linked to a ubiquitous data acquisition network facilitated by a myriad of sensing systems, ranging from wearable devices and Integrated Industry 4.0 manufacturing lines to remote environmental monitoring platforms. Market forecasts indicate that the global sensor sector has entered a phase of exponential growth, driven by advancements in the Internet of Things (IoT), point-of-care medical diagnostics, and quantum metrology [1].

However, as devices trend towards extreme miniaturization and enhanced detection limits, engineering faces a fundamental material-centric bottleneck that cannot be circumvented through signal processing optimization alone. Gold occupies a central position in addressing these challenges. Owing to its unique physicochemical properties—notably high electrical conductivity, exceptional chemical inertness in corrosive environments, and inherent biocompatibility—gold has emerged as a critical functional substrate in the architecture of next-generation sensing technologies.

A review of the current state of the art in important applications such as Resistance Temperature Detectors [2,3,4,5], self-assembled monolayer/surface plasmon resonance, biosensing platforms [6,7,8,9,10], Surface-Enhanced Raman Spectroscopy [11,12,13,14,15], and strain sensors indicates that conventional metallic thin-film fabrication techniques have reached their fundamental physicochemical limits. Depending on the specific application and fabrication technique, several critical limitations of gold-based materials must be addressed. These include susceptibility to contamination and corrosion, suboptimal mechanical strength of the resulting interconnects, and the inherent difficulty of removing stabilizing agents. Furthermore, challenges such as poor adhesion of the deposited layers, the toxicity of constituent components, and the detrimental impact of surfactants/additives on overall conductivity remain significant hurdles in material optimization.

To circumvent these constraints, the integration of gold nanoparticles (AuNPs) via advanced deposition methodologies, such as Ultra-Precise Dispensing (UPD), has emerged as a pivotal technological frontier. The utilization of high-concentration (above 90% (*w*/*w*)) Au-nanoparticle-based pastes within the UPD framework enables precise architectural control over the active sensing layers, directly addressing critical challenges related to interfacial adhesion, signal reproducibility, and long-term structural stability in next-generation sensing devices. The aforementioned additive manufacturing technique facilitates a significant enhancement in both mechanical integrity and purity, while streamlining the sensor fabrication process. By optimizing the architecture of the active layers, this approach ensures superior electrical stability and heightened sensitivity. Beyond general microelectronics, this architectural control is particularly crucial for electrochemical and capacitive biosensing platforms. In these specific applications, maximizing the probe density per unit area is vital. The nanostructured morphology of the paste intrinsically enhances the effective electroactive surface area, thereby facilitating the immobilization of a higher density of capture molecules (e.g., DNA) compared to planar layers deposited via conventional thin-film techniques [7,10,11].

The Au90 nanopaste formulation has been specifically engineered for the UPD process, exhibiting high viscosity and a high metallic packing fraction post-sintering. Extended rheological characterization of the material is currently ongoing. In this study, the primary focus is placed on assessing rheological behavior specifically tailored for the Ultra-Precise Dispensing (UPD) process. Preliminary evaluation of these flow parameters under printing conditions ensures the necessary material stability during and after deposition, providing a functional baseline for resolution. A more fundamental analysis of the linear viscoelastic region (LVR) will be presented in future work as part of the continued development of this novel gold nanopaste. These optimized parameters ensure high material stability both pre- and post-deposition. Consequently, this approach addresses a persistent challenge in gold electrode fabrication: achieving robust interfacial adhesion. Furthermore, the high metal-loading concentration effectively mitigates the adverse effects of substrate surface roughness on the structural integrity of the deposited films. Owing to its optimized rheological and physicochemical properties, the developed Au90 nanopaste demonstrates versatile applicability, enabling its utilization in alternative deposition techniques and across a diverse range of industrial requirements. The unique structural hierarchy and surface energy profile of the synthesized gold nanoparticles in paste enable versatile post-deposition processing, facilitating consolidation not only through conventional thermal annealing but also via advanced non-thermal physical sintering mechanisms. This multi-modal sintering capability—including photonic, plasma, or electrical triggering—circumvents the thermal budget constraints of sensitive organic substrates, providing a universal platform for high-performance flexible electronics. The material presented herein serves as a technological bridge between conventional metallic pastes—prioritizing exceptional electrical conductivity and corrosion resistance—and emerging hierarchical composites that enable the next generation of carbon-fiber and graphene-based flexible strain sensors [16,17]. Furthermore, integrating the high-resolution capabilities of UPD-printed Au90 metallic structures with advanced shielding materials represents a potential paradigm shift in the fabrication of high-performance sensing platforms [18].

A significant advantage of the proposed material lies in the utilization of nanoparticles synthesized via a novel, additive reductant-free protocol. By optimizing kinetic parameters—specifically through the precise control of the nucleation-to-growth ratio via the strategic selection of ligands and stabilizers—a robust synthesis method for pseudo-spherical nanoparticles has been established. The resulting colloidal system exhibits long-term stability (up to six months in the printing cartridge, not only in the syringe), facilitating its versatile application across a wide range of nanomaterial systems. Gold nanoparticles synthesized via the newly developed method facilitate rapid material transfer and integration with diverse deposition technologies, e.g., direct writing and laser deposition. Furthermore, this approach ensures the availability of high-quality nanostructures specifically tailored for advanced functionalization, meeting the rigorous demands of versatile sensor architecture development.

## 2. Materials and Methods

### 2.1. Au90 Nanopaste/Formulation

**Figure 1:**
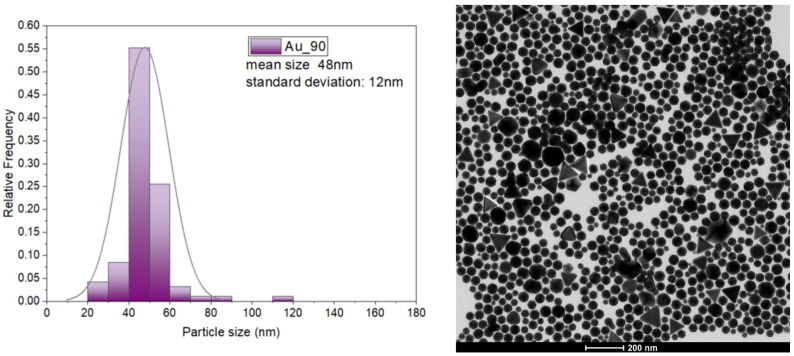
(**Left**) Size distribution, and (**Right**) illustrative TEM image of gold nanoparticles in Au90 nanopaste.

**Figure 2:**
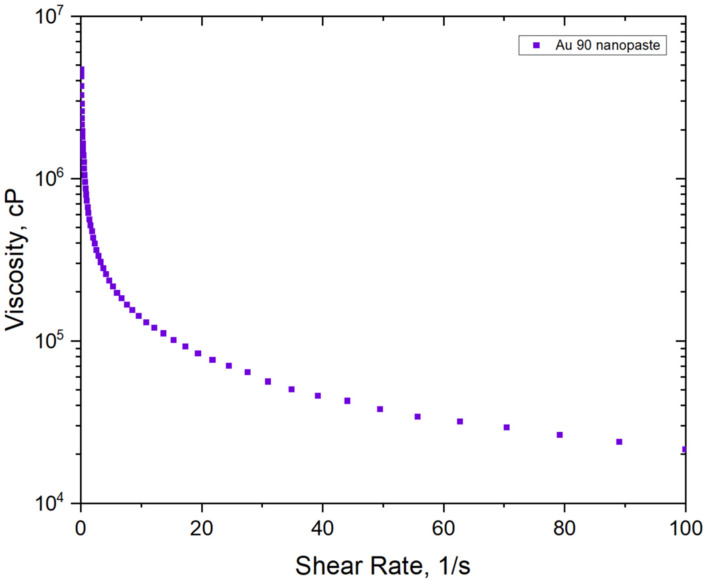
Viscosity as a function of the shear rate for Au90 nanopaste.

The conductive architecture was fabricated using Au90 nanopaste material (XTPL S.A., Wrocław, Poland), a proprietary, high-viscosity metallic ink specifically engineered for advanced sensing applications. The material consists of gold nanoparticles dispersed in a complex organic vehicle. This vehicle includes a mixture of high-boiling-point organic solvents to prevent nozzle clogging and ensure stable flow. To achieve the desired behavior, the formulation incorporates polymeric binders (for structural integrity of the printed trace) and surface active agents (surfactants) to prevent uncontrolled agglomeration. The ink fabrication process follows a systematic streamline: (1) controlled synthesis of gold nanoparticles, (2) two-stage nanoparticle purification, (3) phase transfer into the organic carrier, (4) mechanical homogenization. This nanopaste is characterized by an exceptionally high metallic loading of 90 wt.% (solid content ranging between 87 and 93 wt.%), consisting of ultra-pure gold nanoparticles with a primary size distribution centered between 35 and 55 nm with a pseudo-spherical shape, as verified by transmission electron microscopy (TEM). Figure 1 shows the size distribution along with an illustrative electron microscope image. The resulting micrographs were processed and quantified using ImageJ 1.54gsoftware. To ensure statistical significance, a minimum of 100 individual nanoparticles were measured from multiple representative areas. The image processing workflow involved the following steps: scale calibration, pre-processing, thresholding, and quantification. In contrast, Dynamic Light Scattering (DLS) measurements indicate an effective hydrodynamic diameter of 80–130 nm, with a highly favorable polydispersity index of approximately 0.073, suggesting the formation of stable, soft-agglomerated clusters within the organic vehicle. Table 1 summarizes the most important properties of the Au90 nanopaste material. To ensure high-resolution deposition and structural integrity, the formulation exhibits a distinct non-Newtonian rheological profile, with a dynamic viscosity exceeding 1 × 10^5^ mPa·s at low shear rates, as shown in Figure 2. Upon thermal processing at 350 °C for 20 min in ambient atmosphere, the sintered tracks demonstrate excellent electrical performance, reaching a low bulk resistivity of 8.13 µΩ·cm [19].

This high-viscosity framework is optimized for UPD, facilitating the formation of dense conductive traces with minimal thermal budget requirements. To achieve robust steric stabilization of the gold nanostructures, a novel, internally designed modification of the polyol synthesis was implemented. The resulting colloidal suspension underwent rigorous purification via controlled precipitation to eliminate residual precursors and secondary by-products. In the final stage, the material properties were precisely engineered by introducing a synergistically compatible system of organic solvents and stabilizing agents, specifically tailored to ensure long-term colloidal integrity and optimal rheological performance for the paste.

### 2.2. Printing of High-Density Fractal Structures Using Gold Nanopaste

These microfabrication tasks on high-density fractals were performed using the UPD technology in a Delta Printing System (DPS). The Au90 nanopaste was dispensed at a controlled pressure of 3000 mbar with a translation speed of 0.20 to 0.25 mm/s. Initial results showed linewidths ranging from 5 to 13 µm on glass and 8 to 15 µm on Kapton (h = 200 µm). Following dispensing, the structures were thermally sintered on a hotplate at 350 °C for 20 min.

### 2.3. Temperature Sensor

Prior to printing, the glass substrate (soda-lime glass slide 76 mm × 25 mm × 1 mm, Chemland, Stargard, Poland) was cleaned by an initial rinse with acetone followed by demineralized water, then subjected to ultrasonic cleaning in a mixture of isobutanol and rokafenol (PCC Rokita, Brzeg Dolny, Poland) (80:1 ratio) for 15 min. After a subsequent flush with DI water, the slide underwent further ultrasonic cleaning in a water bath for 15 min, followed by a 20 s immersion in water at 80 °C. The substrate was finally dried using compressed nitrogen.

The Au90 nanopaste was dispensed using the UPD system onto a glass slide at a pressure of 2000 mbar and a translation velocity of 1 mm/s. The opening of the nozzle had a 10 µm outside diameter.

The pattern was sintered at 350 °C for 20 min on a hotplate. The resultant linewidth (after sintering) varied from 15 to 18 µm, with a height of ~1 µm.

Jumper wires were mounted to the microscope glass slide near the lines leading from the printed pattern. An electrical connection was created with solder paste T7-type NP303-DPF201-T7 (NIHON GENMA MFG. CO., LTD., Tokyo, Japan). The assembly was heated at 215 °C for 2 min to facilitate electrical bonding. The printed pattern was encapsulated using a Chemland cover glass coated with Norland Optical Adhesive 81 (NOA 81, Norland Products Inc., Jamesburg, NJ, USA). Structural integrity was secured via photocuring for 2 min using a multi-lamp irradiation chamber equipped with four 9 W low-pressure mercury-vapor emitters (36 W total power). A secondary application of NOA 81 was applied to the solder joints and photocured for an additional 2 min to reinforce the connection points.

Resistance in time was recorded with a KEYSIGHT 6 ½-Digit Multimeter. The total time interval between measurements was 600 ms. Temperature response was detected with a PicoLog TC-08 high-resolution thermocouple data logger with a 200 ms time interval. A type K thermocouple was mounted with tape to the surface of the sensor device. Preliminary performance was verified by monitoring the sensor’s response to thermal stimulation.

## 3. Results

### 3.1. Printing of High-Density Fractal Structures Using Gold Nanopaste

The implementation of deterministic fractal geometries through UPD technology provides a robust framework for exploring hard–soft material integration in microelectronics. In this study, we evaluated five distinct motifs—Gosper, Hilbert, Peano, Sierpinski, and a Double Spiral—to investigate their space-filling properties and mechanical adaptability. These patterns, confined to a 3 mm × 3 mm footprint, allow for a high packing density of conductive traces, which is essential for device miniaturization. By utilizing self-similar layouts, these structures incorporate spring-like motifs across multiple scales, offering enhanced elastic strain capabilities compared to traditional periodic serpentines [19,20,21]. This mechanical resilience is particularly relevant for epidermal electronics, enabling devices to conform to the curvilinear and dynamic surfaces of biological tissues [22].

**Figure 3:**
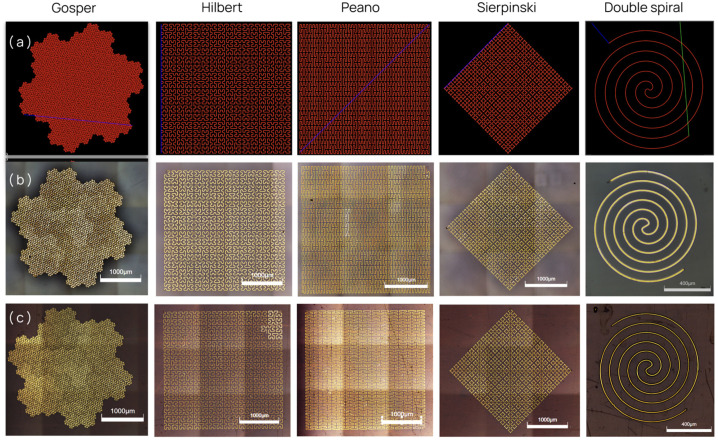
Fabrication and morphological characterization of fractal-based designs via UPD: (**a**) Digital representations of the Gosper, Hilbert, Peano, Sierpinski, and Double Spiral patterns in render mode on UPD. (**b**) Optical images of the gold traces printed on glass substrates (Double Spiral footprint dimension: 1 mm × 1 mm; others: 3 mm× 3 mm). (**c**) Corresponding gold structures printed on flexible Kapton foil. Scale bars: 1000 µm (400 µm for Double Spiral). Samples (**b**,**c**) after the sintering process.

**Figure 4:**
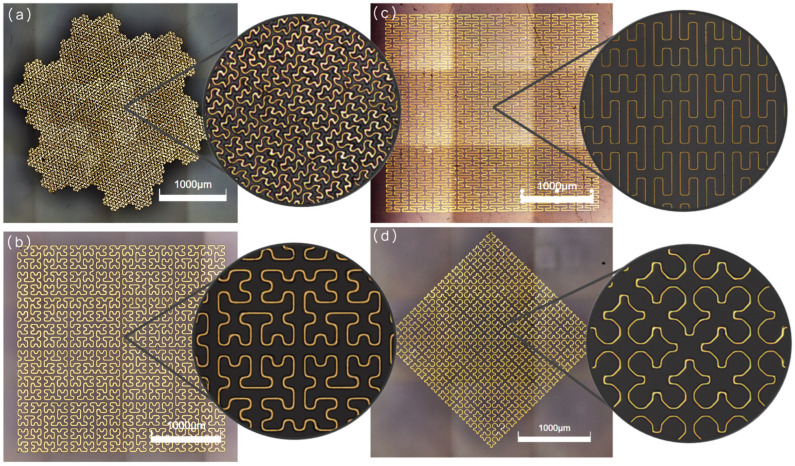
Optical images showing the magnified details of printed fractals using UPD technology: (**a**) Gosper fractal with linewidth of 11 µm printed on glass substrate. (**b**) Hilbert fractal with linewidth of 17 µm printed on glass substrate. (**c**) Peano fractal with linewidth of 8 µm printed on Kapton foil. (**d**) Sierpinski fractal with linewidth of 7 µm printed on glass substrate.

The selection of these specific topologies was guided by their unique geometric and functional advantages. The Hilbert and Peano curves ensure highly uniform surface coverage, while the Gosper and Sierpinski motifs provide hierarchical connectivity that enhances robustness against localized structural defects. Furthermore, the absence of closed loops in those layouts minimizes RF-induced eddy currents, potentially offering invisibility under magnetic resonance imaging [20]. The Double Spiral motif was included to explore the feasibility of printing inductive components and antenna-like structures, building upon established fractal antenna design concepts [23]. This study focuses on the evaluation of the printing process and the characterization of the paste’s behavior during complex path dispensing. To quantify printing performance, a systematic statistical analysis of linewidths and heights was performed across all motifs (measured at *n* = 5 locations per sample). On rigid glass substrates, high morphological fidelity was achieved with linewidths of 5 ± 1 µm for Peano, 7 ± 1 µm for Hilbert, and 9 ± 1 µm for Sierpinski and the Double Spiral. The complex Gosper curve, involving the highest density of directional changes, resulted in a width of 13 ± 2 µm. Corresponding average trace thicknesses on glass were determined via confocal microscopy as follows: Peano (0.6 ± 0.2 µm), Hilbert (1.2 ± 0.1 µm), Sierpinski (1 ± 0.1 µm), and Gosper (1.3 ± 0.3 µm). On flexible Kapton foil, linewidth results remained consistent: Peano (8 ± 1 µm), Hilbert and Gosper (9 ± 1 µm), Sierpinski (10 ± 1 µm), and Double Spiral (15 ± 2 µm). Figure 3 and Figure 4 show various patterns printed using the UPD technology described above. The sharp edge definition and minimal lateral spreading resulted from the high solid content (90 wt.%) and high viscosity (1–2 million cP) of the Au90 nanopaste. This prevented trace deformation post-deposition, maintaining 5 µm features, as shown in Figure 4. Reproducibility was confirmed through dual designs for each motif on glass with varying iteration orders (e.g., max. Hilbert order 6 and Peano order 4), showing consistent morphology and continuity. Under optimized parameters, electrical conductivity was achieved for various samples.

**Figure 5:**
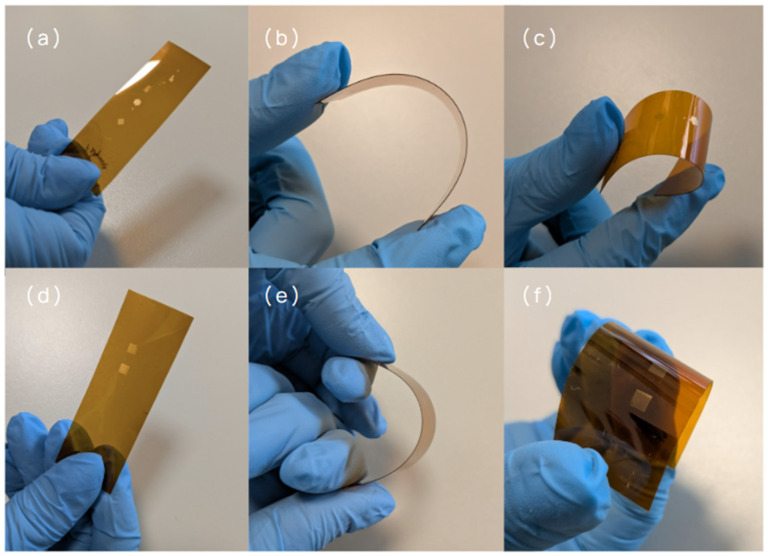
Mechanical reliability and bending performance of fractals on Kapton: (**a**,**d**) Flat state of printed fractal samples on polyimide strips. (**b**,**c**,**e**,**f**) Samples under manual bending, illustrating the structural integrity and macroscopic flexibility of the gold nanopaste traces.

The initial mechanical behavior of the printed traces was assessed through a sequential bending test performed specifically on the Double Spiral motifs on Kapton substrates (h = 200 µm). The resistance was monitored over 90 manual cycles using decreasing bending diameters: 20 mm, 10 mm, and 3 mm, see example in Figure 5. The resistance of the Spiral fractal remained stable during the 20 mm and 10 mm cycles, with initial values of approximately 82.7 Ω, shifting to only 83.4 Ω after 60 cycles. A measurable increase in resistance was observed only when the bending diameter was reduced to 3 mm, reaching 97.4 Ω after 90 cumulative cycles. Despite the resistance increase at the 3 mm limit, optical inspection confirmed that the gold traces maintained continuity without macroscopic delamination. Interfacial adhesion was further assessed via a tape-peel test; the resistance measured after the test was 82.6 Ω, indicating no loss of conductive material. These observations provide an early-stage functional baseline for the reliability of UPD-printed gold fractals on polyimide.

### 3.2. Temperature Sensor

**Figure 6:**
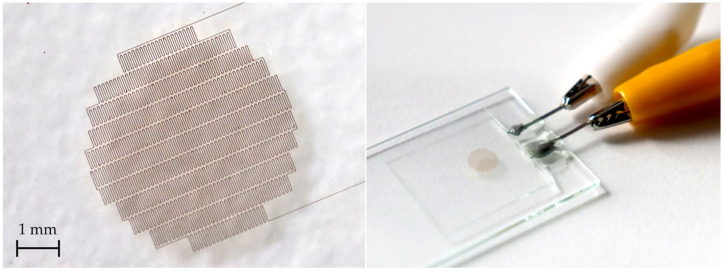
Photographs of the printed temperature sensor: (**Left**) detailed view of the active area, and (**Right**) photograph of the device connected for characterization.

**Figure 7:**
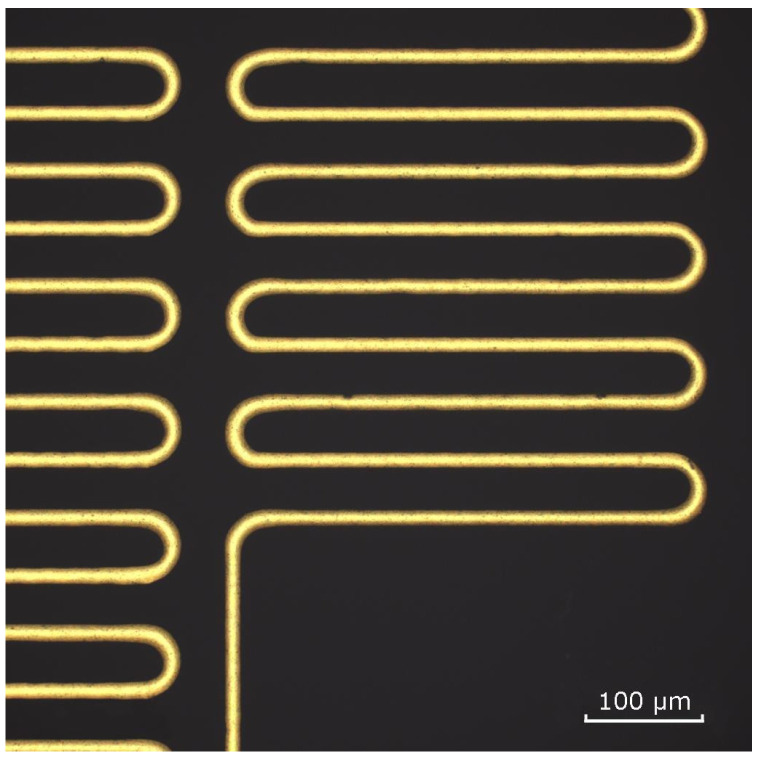
High-magnification optical image of the sensor track. Scale bar: 100 μm.

To demonstrate the practical utility of the Au90 nanopaste, a proof-of-concept temperature sensor was fabricated (as shown in Figure 6 and Figure 7), consisting of a serpentine resistor pattern with a total line length of 39.6 cm. The active area was designed to fit within a 5 mm diameter circle.

**Figure 8:**
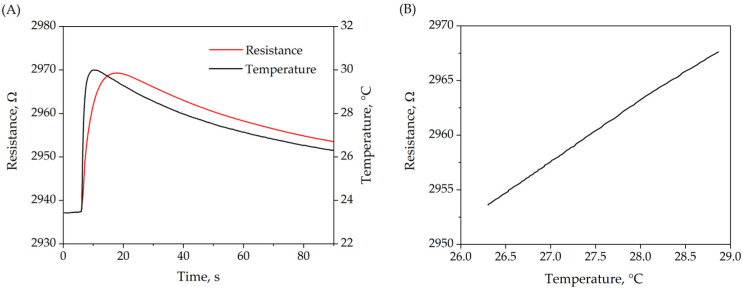
(**A**) Transient resistance and temperature measurements of the gold sensor over the duration of the heating experiment. (**B**) Ohmic response as a function of temperature.

Localized sensitivity of the sensor near room temperature was investigated, as shown in Figure 8A, which illustrates the rapid resistance change upon contact with a pre-heated fingertip. There is a clear resistance response to the change in temperature. The temperature dependence of the resistance was further characterized within the relaxation interval of 25–90 s (Figure 8B), providing the basis for subsequent calculations. For the purpose of data analysis and further calculations, the measured resistance of 2957.6 Ω at 27 °C was utilized as the reference baseline (R_27_). By using this local reference point, the statistical robustness of the derived parameters is maintained across the measured temperature range.

## 4. Discussion

The logic behind choosing fractal geometries for this study, as opposed to standard Euclidean shapes—such as solid rectangles or simple grids—is rooted in their extraordinary space-filling properties and unique mechanical behavior. Fractals are defined by mathematical iterations that produce self-similar patterns—where the structural geometry remains consistent across multiple length scales. This property represents an engineering breakthrough for miniaturization; as demonstrated by Herbko and Łopato [24], such designs allow for a four-fold reduction in sensor area with only a two-fold decrease in sensitivity, a trade-off that is unachievable with traditional planar electrodes. Further research by Herbko et al. [25] suggests that transitioning to fractal resonators can result in a footprint reduction of up to 80% without shifting the operational frequency. In our work, we pushed these boundaries even further by utilizing exceptionally high iteration orders: the sixth order for the Hilbert curve and the fourth order for the Peano curve. The resulting complexity, consisting of thousands of individual vector segments, reached the computational limits of standard CAD environments, highlighting the significant processing task managed by the UPD platform during real-time trajectory execution.

From a process engineering standpoint, the technical simplicity and “one-step” nature of UPD technology offer a decisive advantage over other additive and subtractive methods. Conventional cleanroom microfabrication (CVD/PVD combined with photolithography) is notoriously laborious, requiring expensive masks, hazardous etchants, and capital investments often exceeding USD 1 million [26]. While Aerosol Jet Printing serves as a maskless alternative, it frequently suffers from “overspray” and requires complex sheath gas management to maintain focus. Traditional inkjet printing is further limited by the necessity for very low-viscosity inks, which typically have low metal loading and require multiple layers to achieve functional conductivity [22,26]. In contrast, our UPD approach utilizes a Au90 nanopaste with 90 wt.% metal content. This allows for the deposition of dense, high-aspect-ratio traces in a single pass. Although UPD technology is capable of reaching a resolution of 1 µm [27], we deliberately utilized linewidths of 5–13 µm in this study as a strategic engineering trade-off to ensure maximum process yield and line continuity across the most intricate sections, such as the rapid directional changes in the Gosper curve.

The Gosper curve (fifth order) served as the “stress test” for the UPD system’s stability. Due to its high density of sharp turns and the need for constant, rapid changes in the printing head’s trajectory, it represents the most demanding scenario for high-viscosity dispensing. The successful printing of a continuous 13 µm line on glass and 9 µm on Kapton for this motif confirms that the DPS system’s pneumatic control and trajectory execution can handle extreme geometric complexity without nozzle clogging or flow interruption. In terms of mechanical reliability, preliminary tests indicated that the fractal layouts help in distributing stress during deformation. The mechanical stability observed during sequential bending of the Double Spiral pattern highlights the efficacy of the layout in managing multi-scale strain. The near-constant resistance recorded during the initial 60 cycles (at 20 mm and 10 mm diameters) indicates that the sintered gold network remains well within its elastic limit, effectively utilizing the “structure that stretches” strategy.

By replacing sharp mathematical vertices with arc sections, we neutralized stress concentration points that can typically lead to cracking, while also stabilizing the electrical response of the patterns [28]. During deformation, the fractal units exhibit an in-plane rotation—a mechanism likened by Ho et al. [29] to the “motion of scissors”—which dissipates strain and allows the gold traces to endure stresses far beyond the 20% elastic limit of biological tissue [22,29,30].

The selection of gold as the primary material was dictated by its biocompatibility and chemical inertness, which are critical for future biosensors exposed to sweat and environmental moisture. As noted by Zamani et al. [26], gold electrodes with high surface areas and rougher morphologies enhance charge transfer and reduce steric hindrance for bio-functionalization. Functionally, these architectures act as geometric signal amplifiers. The extreme density of vertices triggers a phenomenon known as the lightning rod effect, creating localized regions of high electric field intensity “hot-spots” that significantly increase the capacitive response of the sensors [21]. This enhancement is even more pronounced at higher iteration orders, as confirmed by recent studies on meta-absorbers [31]. By achieving resolutions in the 5–15 µm range, we provide a platform where device performance can be precisely “tuned” by increasing the fractal order—more edges lead to better charge accumulation on the same minimal surface area [21,25]. This work confirms that UPD-printed gold fractals bridge the gap between high-end lithography and scalable additive manufacturing, aligning with the principles of rational design for next-generation bio-materials. To fully contextualize the performance of the proposed Au90 nanopaste/UPD approach, a comparative analysis against current state-of-the-art additive manufacturing techniques is presented in Table 2. As demonstrated, the UPD method uniquely combines ultra-fine resolution (<10 μm) with exceptionally high metal loading, maintaining excellent mechanical flexibility. While traditional inkjet and aerosol jet printing methods typically rely on low-viscosity inks with metal contents ranging from 5 to 25 wt.%, the Au90 nanopaste successfully utilizes a highly viscous (above 100,000 mPa*s) formulation without sacrificing print resolution. In fact, it achieves a highly competitive minimum linewidth of 5 μm, which surpasses the resolution of standard inkjet printing (<20 μm to 95 μm) and bridges the gap toward complex directed self-assembly methods. Furthermore, despite the massive metal concentration, the sintered structures maintain excellent mechanical flexibility, withstanding 360-degree bending on Kapton substrate, which is often a challenge for highly loaded conductive traces. In summary, the combination of high resolution, maximized conductive mass in a single pass, and robust mechanical resilience demonstrates that the UPD method exhibits the highest metal content while maintaining a resolution below 10 µm in comparison to the listed inkjet techniques. These features position the UPD-printed Au90 nanopaste as a highly scalable candidate for next-generation, miniaturized flexible electronics and sensors.

Beyond the structural and mechanical advantages of the fractal designs, the intrinsic electrical reliability of the sintered Au90 nanopaste was validated through the fabrication of a functional prototype device.

**Figure 9:**
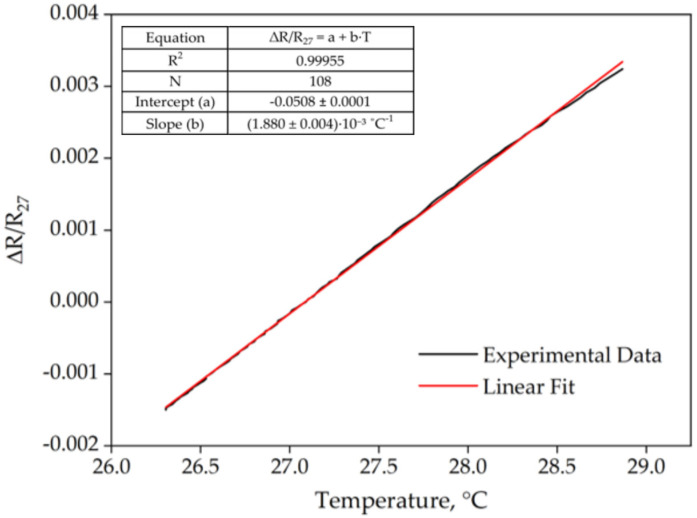
Normalized resistance ΔR/R_27_ as a function of temperature. The slope of the linear fit (red line) represents the temperature coefficient of resistance (TCR).

The electrical performance of the prototype temperature sensor exhibited a robust metallic character within the analyzed temperature range and high sensitivity to thermal fluctuations. A non-linear relationship between resistance and temperature was observed during the initial transient heating phase, attributed to the thermal lag induced by the encapsulation layer. During this period, the system is in a non-equilibrium state where a temperature gradient exists between the external reference and the embedded Au90 nanopaste lines of the sensor. However, in the subsequent relaxation region (25 s–90 s from the measurement start), the system approaches thermal equilibrium. Although the characterized temperature window is narrow (26–29 °C), the high sampling density (N > 100) enabled a statistically significant linear fit to be obtained. The near-unity correlation coefficient (R^2^~0.99955) for the relationship between the relative change in resistance (ΔR/R_270_, where ΔR = R − R_270_) and temperature (Figure 9) indicates that within this range, the Au90 sensor exhibits a highly stable and predictable thermoresistive response. The temperature coefficient of resistance (TCR) was extracted from the slope. The resulting TCR for the sensor was determined to be (1.880 ± 0.004)·10^−3^ °C^−1^ (referenced at 27 °C). This value represents a notably high performance relative to the previously reported inkjet-printed temperature sensors in the literature [40]. Meanwhile, the TCR is approximately 54% of the value for bulk gold (3.5·10^−3^ °C^−1^) [43]. However, the deviation is typical for printed structures due to grain boundaries, porosities, or organic stabilizers that can remain after sintering [43,44].

Furthermore, while minor variations in reported TCR values can also arise from differences in reference temperatures, the performance of the Au90 sensor remains closely aligned with established benchmarks for high-quality printed nanostructures. 

Owing to its simplified architecture and cost-effective fabrication process in this case, the proposed sensor serves as an ideal functional foundation for the advancement of next-generation wearable electronics and flexible monitoring systems.

In this work, the proposed sensor based on a highly concentrated Au90 paste deposited via Ultra-Precise Dispensing (UPD) technology offers a robust alternative to polymer and hydrogel-based systems. While intrinsically stretchable materials, such as the PEDOT:PSS/PUD composite or organohydrogel-based DETO sensors, exhibit remarkably high sensitivities—reaching up to −1.1%/°C and 37.96%/°C, respectively—they rely on thermally activated hopping or ionic migration mechanisms. These mechanisms often result in non-linear Negative Temperature Coefficient (NTC) behavior. In contrast, the Au90 metallic-paste sensor operates on a linear metallic conduction principle. Although metallic sensors typically yield lower temperature coefficient of resistance (TCR) values, they provide superior linearity and predictability, which simplifies signal processing and calibration compared to the complex hopping transport found in reduced graphene oxide (rGO) fibers [45,46]. A significant challenge in wearable electronics is the risk of delamination under cyclic strain. The “lamination-free” approach presented by Makki et al. [47] addresses this through monolithic integration. Our Au90 sensor, utilized with UPD technology, achieves a similar level of structural reliability by enabling high-fidelity printing of tracks as narrow as 7 μm. This precision surpasses the geometric engineering requirements for strain-insensitivity seen in serpentine FSSF fibers, which require manual sewing or embedding processes. The ability to print fractal geometries directly onto flexible polyimide or glass allows for a higher device density than the 3 × 3 or 4 × 4 arrays demonstrated in the comparative studies. A critical drawback of hydrogel-based sensors is their susceptibility to dehydration and humidity, requiring complex encapsulation strategies like the DETO architecture to maintain stability [48]. Similarly, PEDOT:PSS-based sensors are known to be hygroscopic. The Au90 metallic structures are inherently stable under varying humidity levels, without the need for sophisticated water-retention systems. Furthermore, the metallic sensor could provide a much broader sensing range compared to the operational limits of PEDOT:PSS (50 °C) or organohydrogels (95 °C). This makes the Au90-UPD platform particularly suitable for industrial or healthcare environments, where sensors might be exposed to extreme temperatures or high sterilization requirements. In summary, while polymer and hydrogel sensors offer “ultrasensitivity” for specialized soft-robotic applications, the Au90-based sensor fabricated via UPD technology provides a superior combination of linearity, long-term stability, and manufacturing precision, fulfilling the rigorous demands for reliable wearable medical diagnostics. Table 3 provides a detailed comparative analysis between the high-resolution Au90 metallic sensor and alternative platforms.

The temperature resolution of the Au90 sensor was evaluated based on the TCR and the electrical noise floor of the measurement setup. Given a resistance resolution of 0.1 Ωand a TCR of 0.188%/°C, the calculated thermal resolution is approximately 0.02 °C.

The response time of the encapsulated Au90 sensor, defined as the time to reach 90% of the steady-state resistance change (t_90), was determined to be 6 s. This response speed is governed by the thermal inertia of the soda-lime glass substrate and the NOA 81 encapsulation layer. Given that the heat must diffuse through the protective polymer coating before reaching the printed gold tracks, a 6 s response is consistent with expectations for robustly packaged, microscale thermal demonstrators. It should be noted that the reported 6 s response time represents the total system response under a transient thermal load (human fingertip). Unlike a constant-temperature source, the fingertip undergoes natural cooling upon contact with the glass substrate. Therefore, the observed response time is a convolution of the sensor’s intrinsic heating rate and the thermal decay of the source.

Building upon these promising results, future work will focus on derivative ink formulations based on the aforementioned gold nanostructures. These are currently being developed and validated for compatibility with alternative deposition methods, such as Laser-Induced Forward Transfer (LIFT) technology within the framework of the European ULTRA-SOUND with Bioimpedance Analysis and Graphene FET-enhanced Wearable Sensing For Decentralized Health Monitoring project (HORIZON-CL4-2023-RESILIENCE-01-33). This endeavor aims to introduce a novel concept of a stretchable multi-sensing platform for advanced wearable body composition analysis.

## 5. Conclusions

We report on a high-concentration gold nanoparticle (AuNP) architecture, synthesized via a refined polyol-mediated route, exhibiting bespoke rheological properties designed to bridge the gap between laboratory synthesis and industrial-scale integration. This material paradigm addresses the critical bottlenecks currently hindering the evolution of electronic and bioelectronic sensing platforms. When coupled with the XTPL UPD system, this framework enables the fabrication of electrodes with unprecedented feature resolution, facilitating extreme miniaturization across a diverse range of substrate geometries. Furthermore, the inherent versatility of this green-chemistry-based synthesis allows for precise iterative tuning to ensure compatibility with established microelectronic deposition standards. These high-purity AuNPs not only serve as robust sensing transducers but also provide a pristine scaffold for advanced molecular functionalization, offering a sustainable and scalable pathway for next-generation sensor development. The successful deposition of intricate fractal patterns serves as a critical validation of the UPD process, establishing a robust technological pathway for the fabrication of high-performance electrodes with complex topologies. As a proof-of-concept, a microscale temperature sensor was fabricated, exhibiting a TCR of (1.880 ± 0.004)·10^−3^ °C^−1^ (referenced at 27 °C). While this demonstrator was characterized within a localized thermal window, its performance validates the successful integration of material synthesis and high-resolution printing. Future work will extend these capabilities toward more comprehensive investigations, including characterization of thermal drift and hysteresis under controlled conditions to fully evaluate long-term sensor performance.

## Figures and Tables

**Table 1:** Characteristic values for Au90 nanopaste material.

Paste	Solid Content	Metal Content (wt.%)	Mean Nanoparticle Size [nm] (TEM)	Average Nanoparticle Size [nm] (DLS)	Electrical Resistivity [µΩ·cm]	Viscosity (25 °C) [mPa·s]
Au90 nanopaste	87–93	90	35–55	80–130	8.13 (350 °C; 20 min; Air)	>100,000 (Shear Rate = 0.2 s^−1^)

**Table 2:** Comparative overview of rheological, morphological, and electro-mechanical properties of AuNP-based formulations alongside specific processing conditions.

Printing Methods	Materials	Viscosity (mPa·s)	Metal Content (wt.%)	Min. Linewidth (µm)	Substrate and Sintering Conditions	TCR (10^−3^ °C^−1^)	Mechanical Characteristics	Ref.
Ultra-Precise Dispensing	Au90 Nanopaste	>100 000	>90	5	Kapton substrate	1.88	ΔR/R_0_ ≈ 2–5%	[This work]
Inkjet Printing	PVP-stabilized AuNPs suspended in a mixture of H_2_O, EtOH, and EG	1–16	5	~100	Kapton substrate	n.a.	No cracks	[32]
Aerosol Jet Printing	PVP-stabilized AuNPs suspended in a mixture of H_2_O, EtOH, and EG	1–16	5	15–20	Kapton substrate	n.a.	No cracks	[32]
Inkjet Printing	Octanethiol-functionalized AuNPs (OT-AuNPs) with TrisSH dispersed in terpineol	-	25	~95 ± 5	PEN substrate	n.a.	Stability for 1000 cycles (r = 0.6 cm)	[33]
Drop-on-Demand Inkjet Printing	AuNP ink JG-125 (commercial)	-	-	35 (drop spacing)	MTI alumina substrate	2.7	Rigid substrate	[34]
Inkjet Printing	PVP-capped AuNPs in H_2_O/Diethylene glycol/glycerol mixtures	-	11	20	Soda-lime glass	n.a.	Rigid substrate	[35]
Inkjet Printing	Aqueous AuNPs capped with PVP40	2.5–5.8	0.03–0.12	-	Flexible photo paper	n.a.	No cracks after 25× repetitions in continuous distribution of the AuNP clusters on the paper substrate	[36]
Inkjet Printing	AuNPs protected by PVP and acrylic resin in H_2_O and EtOH	~1–3	20	100	Silicon, glass, paper, and flexible projection film	n.a.	Adhesion tests	[37]
Directed Self-Assembly/Bar Coating	π-junction AuNP ink	-	15–25	0.6	Cyclic olefin polymer substrate	n.a.	No cracks	[38]
Flexographic Printing	PVP-capped AuNPs dispersed in 70% IPA/30% H_2_O	-	-	100–120	Polyimide substrate	n.a.	Operation on flexible substrate	[39]
Inkjet Printing	1. AuNP ink: 35% wt. AuNP solution, 55% wt. glycerol, 10% wt. propan-2-ol; 2. Precursor ink: HAuCl_4_ in 20% wt. H_2_O, 70% wt. ethylene glycol, 10% wt. Propan-2-ol	11.2 for 1st ink 14.0 for 2nd ink	5 for 1st ink 20 for 2nd ink	~37	Polyimide foil	n.a.	Bending tests (qualitative)	[40]
Plasma Jet Printing	PVP-stabilized AuNPs synthesized via USP and redispersed in EtOH	~50–70	0.025	550	Al_2_O_3_ technical ceramic substrates	n.a.	Rigid substrate	[41]
Inkjet Printing	AuNPs stabilized in sugar-based biodegradable comb-like polyurethane polymer matrix	1.9–2.1	1.5–3.0	35	Glossy photo paper	n.a.	No cracks	[42]

**Table 3:** Comparison of the sensing performance and fabrication characteristics of the Au90 temperature sensor with recently reported state-of-the-art thermal sensors.

Parameter	PEDOT:PSS/PUD	FSSF (rGO/PU)	DETO (Organohydrogel)	Sensor PoC from This Article
Sensing Material	PEDOT:PSS/polyurethane dispersion	rGO/polyurethane composite	PAM/carrageenan double-network organohydrogel	Au90 nanopaste
Sensitivity (TCR)	−1.1%/°C	0.8%/°C	37.96%/°C	0.188%/°C
Resolution	0.1 °C	0.1 °C	Not explicitly stated	0.02 °C
Sensing Range	25–50 °C	30–80 °C	25–95.7 °C	26–29 °C
Response Time	8.5 s	7 s	6.01 s	6 s
$\mathrm{Linearity}\;(R^{2}$)	~0.98	Not explicitly stated	Not explicitly stated	~0.99
Ref.	[47]	[46]	[48]	[This work]

## Data Availability

The datasets generated and analyzed during the current study are not publicly available due to confidential.

## Abbreviations

The following abbreviations are used in this manuscript:

UPD	Ultra-Precise Dispensing
IoT	Internet of Things
SPR	Surface plasmon resonance
SERS	Surface-Enhanced Raman Spectroscopy
AuNPs	Gold nanoparticles
LVR	Linear viscoelastic region
FDA	Food and Drug Administration
XTPL S.A.	XTPL Spolka Akcyjna
TEM	Transmission electron microscopy
DLS	Direct Light Scattering
CVD	Chemical Vapor Deposition
PVD	Physical Vapor Deposition
TCR	Temperature coefficient of resistance
G-FET	Graphene Field Effect Transistors
RF	Radio Frequency
LIFT	Laser-Induced Forward Transfer
